# Supplementary material for: Further Insights on RNA Expression and Sperm Motility
Source: Genes (Basel). 2022 Jul 21;13(7):1291. doi: 10.3390/genes13071291 (PMC9319021; doi:10.3390/genes13071291)
Supplement: Supplementary file 1 [file genes-13-01291-s001.zip › genes-1804620-supplementary.pdf]

Supplementary Table S1. List of primers used to study mRNAs

Primer	Transcript	Sequence	Positive control	TA
ZMYND10-F	NM_015896.4	TGGAGCGAGAAAACAGAGGC	Ciliated cells	60ºC
ZMYND10-R		ATTCTGGCATCGTGAGCAGC		
TEKT2-F	NM_014466.3	AACCATCGCTGCCCTGAAG	Ciliated cells	58ºC
TEKT2-R		CATGGAGTTGGCCTTGCAG		
SPATA33-F	NM_001271907.2	CTGATGTAAAGCAAAAGTCCAGC	PBMC	60ºC
SPATA33-R		GGAACTGCAACTGACTAGCG		
RSPH6A-F	NM_030785.4	CGTGAACGCCCGAAAGATC	Testis	60ºC
RSPH6A-R		CGCCCTCCTCCTCACTAAAC		
CATSPER3-F	NM_178019.3	TCTCAACACCGTCACCAGC	Testis	60ºC
CATSPER3-R		GCATTCGATGTGACAGTGC		
CATSPER2-F	NM_172095.4	CCCTCAAGAGCATGACCTTCC	PBMC	58/60ºC
CATSPER2-R		ACTCTGAGAAGACGTAGACACC		
BSCL2-F	NM_001122955.4	TCCCTGTTGCCAATGTCTCG	Ciliated cells	60ºC
BSCL2-R		AGAAGTGGAGATGATTCGGCC		
IQCG-F	NM_032263.5	ATCCCGGAAACTCTAGAGCC	Ciliated cells	59ºC
IQCG-R		TGATTGTTGAGGCCATTGGAAG		
CFAP43-F	NM_025145.7	AAACGCCGACCAAGGATTTC	Ciliated cells	60ºC
CFAP43-R		AGATCCTGGTAGTTCTCCGAAAG		
PLAG1-F	NM_002655.3	CAACAAGACTGCACCAAGGC	PBMC	59ºC
PLAG1-R		AGTTCTTGCCACATTCTTCGC		
CFAP44-F	NM_001164496.2	GCCTTGGACTGCTCTGTTCG	Ciliated cells	58ºC
CFAP44-R		GCTCCAGTGAAGTTTACCATTCG		
QRICH2-F	NM_001388453.1	CCATCAGGTCAGCACGCT	Testis	60ºC
QRICH2-R		TGCTGGTGGTGATGTTGAGC		
TTC21A-F	NM_001366900.1	CATGAAACTGGACAAGGATGGC	Ciliated cells	60ºC
TTC21A-R		CCTTGCATGCTGGAGAAGTGAAG		
KRT34-F	NM_021013	GCTGACGGAGAGCGAGGCCCAC	Ciliated cells	62ºC
KRT34-R		CCGGGCACGCACGTCCAGCA		
CRHR1-F	NM_001145146	AGCACGCATGTCCCTCCAAGGCTGT	Ciliated cells	62ºC
CRHR1-R		TCACGAGTTGCCCATGATGCCCCA		
LRRC6-F	NM_012472.6	GCCATGGGCTGGATCAC	Ciliated cells	60ºC
LRRC6-R		TGCTGATGCAACGAGAGTTC		
HIP1-F	NM_005338.7	CATGAGAAAGGGGCACAGAC	PBMC	59ºC
HIP1-R		TTCAGGTAGATGCTGCACAG		
CCDC40-F	NM_017950.4	TTAGGCCCGTCGGAGCAAATGG	Ciliated cells	60ºC
CCDC40-R		ACTGGCTCCTGCGAGACGAACT		

TA- anneling temperature

Supplementary Table S1 (continuation). List of primers used to study mRNAs

Primer	Transcript	Sequence	Positive control	TA
DRC1-F	NM_145038.5	GAGCCTTTGATGTGGACAG	Ciliated cells	60ºC
DRC1-R		TCTGTGTGGCGGACTTCTG		
USP11-F	NM_004651.3	CCGCAAGCCAGAGCAGCACC	Ciliated cells	60ºC
USP11-R		TGCGCTCCCGCAGGTAGACA		
GAPDH-F	NM_001289745.2	AGGTCGGAGTCAACGGATTT	-	58ºC/60ºC
GAPDH-R		TGGAATTTGCCATGGGTGGA		
B2M -F	NM_004048	TGGGTTTCATCCATCCGACA	-	58ºC/60ºC
B2M -R		ACGGCAGGCATACTCATCTT		

TA- anneling temperature

Supplementary Table S2. List of the mRNA highest score corresponding target microRNA

mRNA	Highest score corresponding target microRNA
QRICH2	-
LRRC6	miR-3664-5p
RSPH6A	miR-2110
KRT34	-
CRHR1	-
TEKT2	miR-4660
CATSPER3	miR-492
CFAP44	miR-4425
HIP1	miR-4731
IQCG	miR-4514
SPATA33	miR-518c-5p

Supplementary Table S3. List of the primers used for the selected microRNAs

Primer	Sequence	Lengh (BP)
**miR-4660-F**	GCAGCTCTGGTGGAAAATG	19
**miR-4660-R**	GGTCCAGTTTTTTTTTTTTTTTCTC	25
**miR-518c-5p-F**	TCTCTGGAGGGAAGCAC	17
**miR-518c-5p-R**	GTCCAGTTTTTTTTTTTTTTTCAGAAAG	28
**miR-2110-F**	GGAAACGGCCGCTGA	15
**miR-2110-R**	GGTCCAGTTTTTTTTTTTTTTTCACT	26
**miR-492-F**	GACCTGCGGGACAAG	15
**miR-492-R**	GGTCCAGTTTTTTTTTTTTTTTAAGAATC	29
**miR-4514-F**	GCAGACAGGCAGGATTG	17
**miR-4514-R**	GGTCCAGTTTTTTTTTTTTTTTCCC	25
**miR-4425-F**	CAGTGTTGGGATTCAGCAG	19
**miR-4425-R**	GGTCCAGTTTTTTTTTTTTTTTATGGT	27
**miR-572-F**	CCGCTCGGCGGT	12
**miR-572-R**	AGTTTTTTTTTTTTTTTGGGCCA	23
**miR-3664-5p-F**	GCAGAACTCTGTCTTCACTCA	21
**miR-3664-5p-R**	CAGGTCCAGTTTTTTTTTTTTTTTACT	27
**miR-4731-5p-F**	TGGGGGCCACATGAG	15
**miR-4731-5p-R**	GTCCAGTTTTTTTTTTTTTTTCACAC	26
